# Supplementary material for: miR156 Is a Negative Regulator of Aluminum Response in Medicago sativa
Source: Plants (Basel). 2025 Mar 19;14(6):958. doi: 10.3390/plants14060958 (PMC11945701; doi:10.3390/plants14060958)
Supplement: Supplementary file 1 [file plants-14-00958-s001.zip › plants-3486429-supplementary.pdf]

miR156 is a negative regulator of aluminum response in *Medicago sativa*

Gamalat Allam[1, 2], Solihu K. Sakariyahu [1, 2], Tim McDowell [1], Tevon A. Pitambar [1, 2], Yousef Papadopoulos[3], Mark A. Bernards[2], Abdelali Hannoufa[1, 2]

Supplemental Data

Table S1. Primers used for PCR amplification, genotype analyses and qRT-PCR**.** The underline base pairs represent the *attB1*(in the forward primers) and *attB2* (in the reverse primers) recombinant sequences for Gateway® cloning. Asterisks (*) indicate the references genes used for data normalization based on (Alexander et al. 2007).

Name	Sequence (5'-3')	Product size (bp)	Target gene
STTM156F-GW	G GGG ACA AGT TTG TAC AAA AAA GCA GGC TCC AAAAGCCTGAACTCACCGCG	500	*miR156*
STTM156R-GW	GGG GAC CAC TTT GTA CAA GAA AGC TGG GTC TCTTTGTAGAAACCATCGGCG	500	*miR156*
Ms-SPL13Fq1	ACTGCAACCACCTACGAAGA	93	*SPL13*
Ms-SPL13Rq1	AAGAAGAGGTGTGGTTTGGCT	93	*SPL13*
MsSPL13a-Fq1	CTTGGGTTGGAGGAGATGTT	100	*SPL13a*
MsSPL13a-Rq1	GCTGCTGGTTGAAGATGTTG	100	*SPL13a*
Ms-SPL12Fq1	CCTCAGCCTGAAGCAGTGAA	174	*SPL12*
Ms-SPL12Rq1	CTTGCTGTTGGGCATGTCTG	174	*SPL12*
Ms_156Fq3	TGTTCCCATTCATCACCTCCAA	80	*MsmiR156*
Ms_156Rq3	AGGGAGTAGCGGTGATCTTG	80	*MsmiR156*
Acc1-F *	GATCAGTGAACTTCGCAAAGTAC	91	*Acetyl CoA*
Acc1-R *	CAACGACGTGAACACTACAAC	91	*Acetyl CoA*
Acc2-F *	GATCAGTGAACTTCGCAAAGTAC	154	*Acetyl CoA*
Acc2-R *	GAGGGATGCTGCTACTTTGATG	154	*Acetyl CoA*
LA-MsSPL9-Fq1	AGATACAGCTCTTGCTACTG	112	*SPL9*
LA-MsSPL9-Rq1	GTTGAGAATGTTGAACTGAC	112	*SPL9*
Ms-SPL6Fq1	CTCGGCCGATACATCAAACTG	191	*SPL6*
Ms-SPL6Rq1	CCTCTGTTCAACACCATTGACG	191	*SPL6*
MsSPL7a-Fq1	CAGGGCACAATGAGAGAAGA	130	*SPL7a*
MsSPL7a-Rq1	GCCATGAATCAGTCCGAGAT	130	*SPL7a*
MsSPL8-Fq1	TGGCCGCACTTACTTCTCTT	100	*SPL8*
MsSPL8-Rq1	AACCTTCGGCTTGACACCTA	100	*SPL8*
LA-MsSPL4-Fq1	GATGACTCAAAAAGAAGTTG	97	*SPL4*
LA-MsSPL4-Rq1	ATGTCTGAAATCATTCTCCT	97	*SPL4*
MsSPL3-Fq1	GCTTGCAGAGTTTGATGAATCG	112	*SPL3*
MsSPL3-Rq1	GCCAGTACCGCTTCCTTCAG	112	*SPL3*
MsSPL2a-Fq1	GCTGCAGACGACAACTTTCA	100	*SPL2*
MsSPL2a-Rq1	CATCTGTTGCTTCCCATCAC	100	*SPL2*
MsSPL11-Fq1	CTTGTCTTGTGGATGGGTGC	147 bp	*SPL11*
MsSPL11-Rq1	GAAACCTGCTACACTGTTGGC	147 bp	*SPL11*
MsAuxin1-Fq1	GACTACATACACTGCTTGGT	120 bp	*MsAuixn1*

MsAuxin1-FR1	AGTGGCAGAAGGAATCGTTA	120 bp	*MsAuixn1*
MsPIN2-Fq1	GATGCTGGTCTTGGAATGGC	120 bp	*MsPIN2*
MsPIN2-Rq1	ATTGCTATTGAGGTTGCCGC	120 bp	*sPIN2*
MsPG1-Fq1	TATGGAGCAGTTG GAGATGGA	100 bp	*MsPG1*
MsPG1-Rq1	AGAC-GAGATGTGCCTGATTTG	100 bp	*MsPG1*
MsPG4-Fq1	GTGTGGATTGATCGTATTGCTTGTGG	100 bp	*MsPG4*
MsPG4-Rq1	GTCACTGTCACGTTCTCAACTCCTG	100 bp	*MsPG4*

File S1. Python Script for Quantifying Leaf Area Using PlantCV

```
#!/usr/bin/env python

import matplotlib.pyplot as plt

from plantcv import plantcv as pcv

from plantcv.parallel import workflow_inputs

# Get command-line options

args = workflow_inputs()

# Set variables

pcv.params.debug = args.debug

pcv.params.debug_outdir = args.outdir

img, imgpath, imgname = pcv.readimage(filename=args.image1)

img1 = pcv.white_balance(img, roi=(100,250,150,150))

 crop_img = pcv.crop(img=img1, x=25, y=50, h=2825, w=1995)

rotate_img = pcv.transform.rotate(crop_img, 180, False)

pcv.params.debug = "print"

out = args.outdir

thresh1 = pcv.threshold.dual_channels(rgb_img=rotate_img, x_channel="l", y_channel="b", 
points=[(40,80),(125,140)], above=True)

a_fill_image = pcv.fill(bin_img=thresh1, size=80)

a_fill_image = pcv.fill_holes(a_fill_image)
```

```
roi1 = pcv.roi.rectangle(img=rotate_img, x=15, y=275, h=2550, w=1850)

kept_mask  = pcv.roi.filter(mask=a_fill_image, roi=roi1, roi_type='partial')

labeled_objects, n_obj = pcv.create_labels(mask=kept_mask)

marker = pcv.Points(rotate_img, figsize=(6,6))

labeled_objects2, n_obj2 = pcv.create_labels(mask=a_fill_image, rois=roi1, roi_type="partial")

if args.writeimg == True:

    outfile = args.outdir + "/" + filename

pcv.params.debug = "print"

out = args.outdir

############### Analysis ################

analysis_image = pcv.analyze.size(img=rotate_img, labeled_mask=labeled_objects, n_labels=n_obj)

color_histogram = pcv.analyze.color(rgb_img=rotate_img, labeled_mask=labeled_objects, n_labels=n_obj,
colorspaces='hsv')

pcv.outputs.save_results(filename=args.result)

Python software ( version 3.11.5)
```

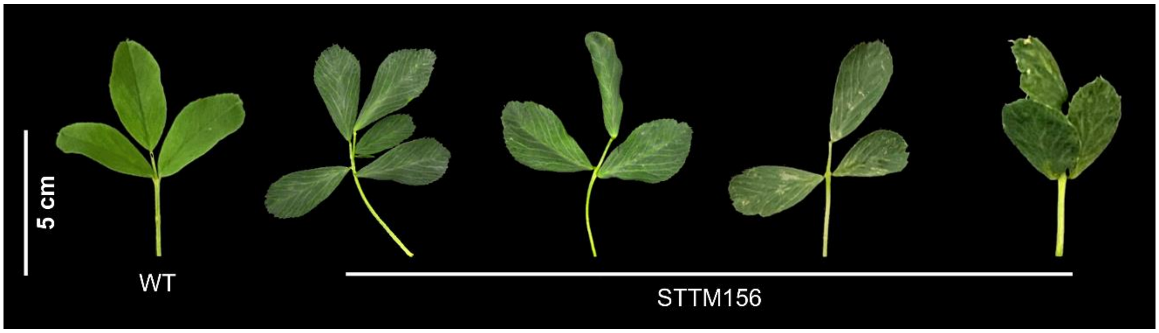

Figure. S1. The effect of silencing miR156 leaf color in MsSTTM156 plants compared to WT. MsSTTM156 leaves show a darker green coloration compared to the lighter green observed in WT. Scale bar = 5 cm.

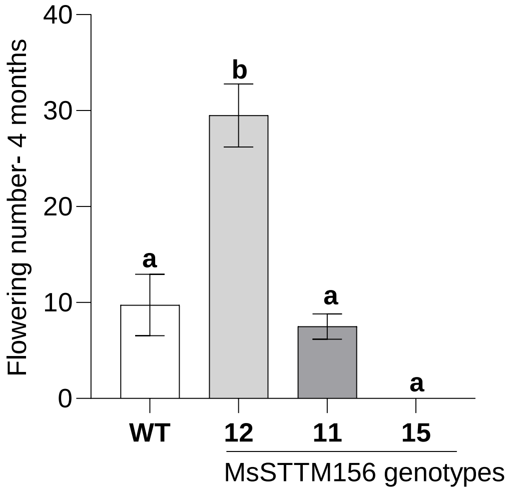

Figure S2. The effect of miR156 silencing/ knockdown (MsSTTM156 genotypes) on flower numbers in four-month-old plants. MsSTTM156-12 and MsSTTM156-11 displayed early flowering, with MsSTTM156-12 showed a significantly higher flower number compared to both MsSTTM156-11 and WT. Each bar plot indicates the mean. Error bars represent SEM. An one-way ANOVA was conducted with n = 4 individual plants. Significant differences detected from the one-way ANOVA in R (version R-4.2.3) tools were followed by a post hoc Tukey multiple comparison test. Means with the same letters are not significantly different at a *p*-value $\leq$ 0.05.

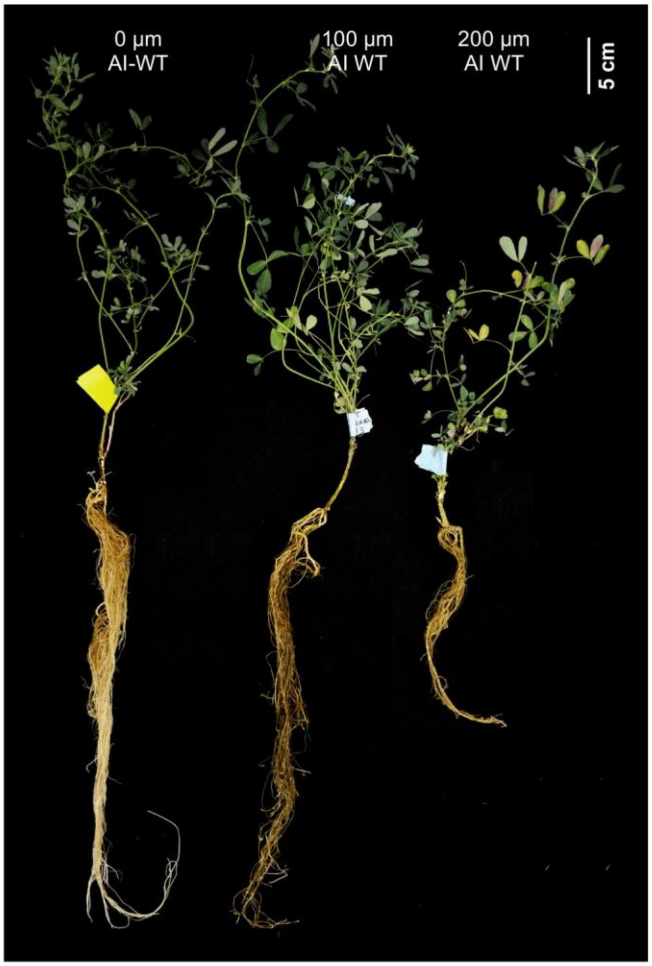

Figure S3. The effect of aluminum concentration on root growth in WT *Medicago sativa*. WT alfalfa plants were grown for 14 days on ½-strength Hoagland's medium (pH 4.5) under three aluminum treatments (0, 100, and 200 μM Al**).** Scale bar = 5 cm.
